# Weeding Out Risk Factors? Study Reports No Association between Prenatal Air Pollution and Autism

**DOI:** 10.1289/ehp.124-A23

**Published:** 2016-01-01

**Authors:** Wendee Nicole

**Affiliations:** Wendee Nicole has written for *Discover*, *Scientific American*, and other publications.

*In utero* exposures to certain toxic constituents of traffic-related air pollution have been linked to neurological impairments. Recent studies have addressed whether exposures to such air toxicants might be environmental risk factors for autism spectrum disorders (ASDs)—developmental disabilities that can involve rigid, repetitive behaviors and impairments in communication and social interactions. Although several past studies reported associations between ASDs and certain air pollutants, a new analysis of four prospective cohorts published in this issue of *EHP* appears to contradict those findings, reporting no such associations.^1^

Working as part of the European Study of Cohorts for Air Pollution Effects (ESCAPE) project,^2^ the researchers analyzed data from four different prospective cohorts investigating ASDs in relation to air pollution. These studies assessed autistic traits, but not formal diagnoses of an ASD, in more than 8,000 children in the general population. The presence of autistic traits had been assessed using validated screening tests, which varied in the included studies. The authors of the current study used land-use regression models to estimate mothers’ at-home exposures to nitrogen oxides (NO_x_) and various sizes of particulate matter (PM) during pregnancy.^1^

**Figure:**
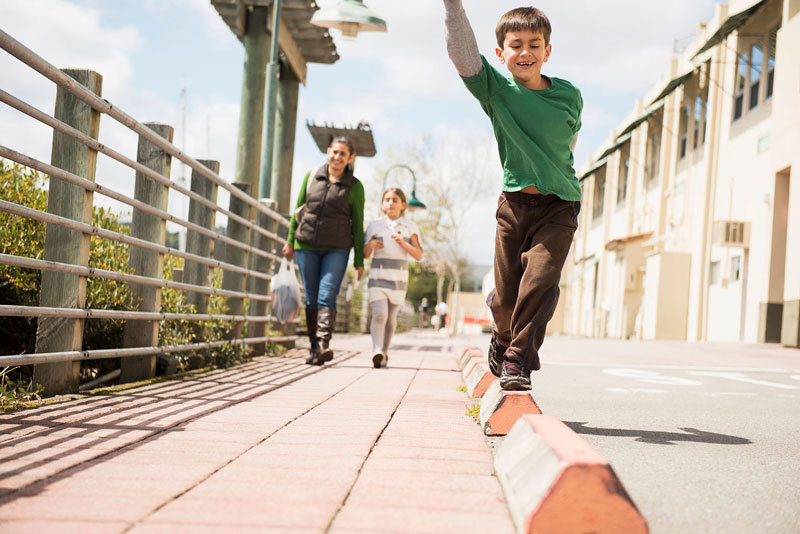
Although several earlier studies reported a higher prevalence of autism in children exposed prenatally to traffic-related air pollutants, a new analysis of four prospective cohorts finds no such association. © Sollina Images/Alamy

Depending on the study, 0.7–3.6% of these children—mostly boys—showed autistic traits in the clinical range, and 3.2–12.3% showed traits in the clinical/borderline range. In the final analysis, no associations were found between presence of autistic traits and prenatal exposure to any of the air pollutants.^1^

“Almost all previous studies carried out mainly in the United States found an association between air pollution and ASDs,” says lead author Mònica Guxens, an assistant research professor at the Centre for Research in Environmental Epidemiology in Barcelona. “I do not think that ASDs are different in Europe than in the U.S., but the best way to check what is going on here is to try to replicate the U.S. studies in Europe following a similar study design.”

Bing-Fang Hwang, a professor of environmental and occupational epidemiology at China Medical University, points out that in three of the studies parents assessed their children’s traits, while psychologists assessed traits in the fourth. “I do not think it’s appropriate to combine all results through meta-analysis without considering the heterogeneity of different diagnoses from parents and a psychologist,” he says. “It may cause bias in estimation of combined odds ratio. The results should be interpreted cautiously.”

The authors acknowledge this and other limitations in their paper. However, the consistent null findings across cohorts suggest their results are not spurious and must be investigated further.^1^

Raanan Raz, an epidemiologist at the Hebrew University of Jerusalem-Hadassah, says the study is important because it examines the issue of air pollution and autism in a new approach—looking at autistic traits in prospective cohorts of children enrolled at birth. He says, “The researchers interpreted [results] with appropriate caution, stating the possible explanations in their discussion.” Among those possible explanations, the authors hypothesize that cohorts of children like the ones in their study would have a vanishingly small proportion of the extreme autism phenotypes that appear in case–control studies.^1^
